# Single-Trait and Multiple-Trait Genomic Prediction From Multi-Class Bayesian Alphabet Models Using Biological Information

**DOI:** 10.3389/fgene.2021.717457

**Published:** 2021-10-11

**Authors:** Zigui Wang, Hao Cheng

**Affiliations:** Department of Animal Science, University of California, Davis, Davis, CA, United States

**Keywords:** multiple-trait, multi-class, genomic prediction, Bayesian Alphabet, biological information

## Abstract

Genomic prediction has been widely used in multiple areas and various genomic prediction methods have been developed. The majority of these methods, however, focus on statistical properties and ignore the abundant useful biological information like genome annotation or previously discovered causal variants. Therefore, to improve prediction performance, several methods have been developed to incorporate biological information into genomic prediction, mostly in single-trait analysis. A commonly used method to incorporate biological information is allocating molecular markers into different classes based on the biological information and assigning separate priors to molecular markers in different classes. It has been shown that such methods can achieve higher prediction accuracy than conventional methods in some circumstances. However, these methods mainly focus on single-trait analysis, and available priors of these methods are limited. Thus, in both single-trait and multiple-trait analysis, we propose the multi-class Bayesian Alphabet methods, in which multiple Bayesian Alphabet priors, including RR-BLUP, BayesA, BayesB, BayesCΠ, and Bayesian LASSO, can be used for markers allocated to different classes. The superior performance of the multi-class Bayesian Alphabet in genomic prediction is demonstrated using both real and simulated data. The software tool JWAS offers open-source routines to perform these analyses.

## 1. Introduction

Genomic prediction, proposed by Meuwissen et al. (2001), utilizes genomic information, such as single-nucleotide polymorphisms (SNPs), to estimate genotypic values or breeding values of complex traits. In the last decades, with the fast development of genotyping and sequencing technology, high-density genotype data has become much easier to access (Harris et al., 2011; Kranis et al., 2013). Accompanied by the high-density data, genomic prediction has been widely used in many areas, including animal breeding (e.g., Hayes et al., 2009a; Erbe et al., 2012), plant breeding (e.g., Wang et al., 2018; Moeinizade et al., 2020), and human disease risk prediction (e.g., Abraham et al., 2014, 2016).

A large number of genomic prediction methods with different statistical assumptions have been developed. Among these methods, genomic best linear unbiased prediction (GBLUP) (Habier et al., 2007; VanRaden, 2008; Hayes et al., 2009b), where a genomic relationship matrix is used to accommodate the covariances among breeding values, is widely used. GBLUP, however, assumes a priori that all marker effects share the same normal distribution, which may not be biologically meaningful, especially for traits controlled by a few causal variants. Furthermore, a collection of Bayesian Alphabet methods (Meuwissen et al., 2001; Fernando and Garrick, 2013; Cheng et al., 2018b; Gianola and Fernando, 2020) have been developed to incorporate different priors on marker effects, e.g., BayesA and BayesB (Meuwissen et al., 2001). Notice that GBLUP is equivalent to a Bayesian Alphabet model with a normal prior for the marker effects (Fernando, 1998; Habier et al., 2007; Strandén and Garrick, 2009). These methods, however, are still developed mainly based on statistical consideration and ignore the abundant biological information. To bridge the gap between the statistical model for genomic prediction and underlying biological architectures, researchers have proposed several methods to incorporate biological information into genomic prediction and have shown that incorporating biological information has the potential to improve the prediction accuracy in some cases (Zhang et al., 2014; Gao et al., 2015; Edwards et al., 2016).

One purpose of incorporating biological information is to relax the assumption that each locus is equally likely to affect the trait, i.e., all loci share the same prior distribution. This assumption is less biologically meaningful, e.g., some loci may be known to lead non-synonymous coding changes or have functional effects on candidate genes (MacLeod et al., 2016). One strategy to achieve this purpose is weighting markers based on the biological information and then integrating the weighting information into the model construction (Zhang et al., 2014; Gao et al., 2015). Zhang et al. (2014) incorporated the QTL list obtained in previous genome-wide association studies (GWAS) into GBLUP, i.e., when constructing genomic relationship matrix, markers were weighted based on the frequency of corresponding genomic regions being reported in the QTL list (Zhang et al., 2014). Gao et al. (2015) incorporated previous GWAS results by using locus-specific inclusion probability based on the *p*-values from GWAS.

In addition to weighting markers, another strategy to incorporate biological information is marker allocation. It has been observed that molecular markers from different genomic regions have different prediction abilities (Erbe et al., 2012; Morota et al., 2014; Do et al., 2015; Abdollahi-Arpanahi et al., 2016) and the marker allocation is beneficial if a particular class is enriched for QTL. To better fit these genomic regions with different genetic architectures, recent studies have tried to allocate genome-wide molecular markers into multiple classes based on the prior biological information and conduct genomic prediction based on these marker classes jointly. Speed and Balding (2014) proposed such a method under the GBLUP framework called MultiBLUP, which divides breeding values into multiple classes to allow different effect-size variances. A Bayesian regression method called BayesRC (MacLeod et al., 2016) was also proposed to allocate SNPs into multiple classes, where a BayesR prior was assigned to each class. It has been shown that allocating markers into different classes can improve predictive accuracy in some circumstances (Speed and Balding, 2014; MacLeod et al., 2016). The idea to allocate markers into multiple classes has also been used in a haplotype-based genomic prediction model (Xu et al., 2020), in which effects of haplotype blocks are estimated using both numerical dosage and categorical coding strategies (Martini et al., 2017) for each genomic class.

To our knowledge, most methods that allocate SNPs into different classes, focus on single-trait analysis and available priors of these methods are limited. Thus, the primary goal of this research is to present a more general Bayesian Alphabet method that can handle both single-trait and multiple-trait analysis, while is able to assign multiple Bayesian Alphabet priors, including RR-BLUP, BayesA, BayesB, BayesCΠ, and Bayesian LASSO, to markers in different SNP classes. The new genomic prediction method we implemented is called multi-class Bayesian Alphabet, where the term “Bayesian Alphabet” denotes a collection of Bayesian Alphabet priors adopted for marker effects. Our multi-class Bayesian Alphabet works for both single-trait and multiple-trait analysis. The performance of the multi-class Bayesian Alphabet is studied using real and simulated data.

## 2. Materials and Methods

### 2.1. Multi-Class Bayesian Alphabet Models

For simplicity, the general mean is assumed as the only fixed effect, thus the general form of the multi-class Bayesian Alphabet model for *i*th genotyped observation can be written as:


(1) $$\begin{array}{c} y_{i}=\mu +\sum_{l=1}^{g}\sum_{f_{l}\in C_{l}}m_{if_{l}}\alpha_{f_{l}}+e_{i} \end{array}$$

where ***y***_*i*_ is a vector of phenotypic values of *t* traits for observation *i*; ***μ*** is a vector of overall means for *t* traits; *m*_*i*_*f*__*l*__ is the genotype covariate at locus *f*_*l*_ (coded as 0,1,2) in SNP class *C*_*l*_ for observation *i*; *g* is the number of SNP classes; ***α***_*f*_*l*__ is a vector of the corresponding allele substitution effects (marker effects) of *t* traits for locus *f*_*l*_; and ***e***_*i*_ is a vector of residuals for observation *i*. Note that when the number of traits *t* = 1, the general form above simplifies to the single-trait model, and all vectors of effects in Equation 1 become scalars. The fixed effect ***μ*** is assigned a flat prior. The residuals, ***e***_*i*_, are a priori assumed to be independently and identically distributed multivariate normal vectors with null mean and covariance matrix ***R***, which is assigned an inverse Wishart prior distribution, $W^{-1}\left(S_{e},\nu_{e}\right)$, with degrees of freedom ν_*e*_ = 4 and scale matrix ***S***_*e*_ such that the prior mean of ***R*** equals half of the phenotypic variance. Note that when number of traits *t* = 1, the prior for ***R*** follows a scaled inverted chi-square distribution.

To incorporate known biological information, marker effects of SNPs in the same class are assumed to have identical Bayesian Alphabet prior. Different from conventional Bayesian Alphabet methods, our multi-class Bayesian Alphabet methods allow assigning different Bayesian Alphabet priors to marker effect ***α***_*f*_*l*__ in different SNP classes. These priors are discussed in the following section 2.2.

### 2.2. Bayesian Prior for Marker Effects

Multiple priors are implemented in our multi-class Bayesian Alphabet models, including BayesA, BayesB, BayesCΠ, RR-BLUP, and Bayesian LASSO. In multiple-trait analysis, with BayesB and BayesCΠ priors, each locus is allowed to affect any combination of traits (Cheng et al., 2018b). In multiple-trait BayesB and BayesCΠ, the vector of marker effects at locus *f*_*l*_ can be written as ***α***_*f*_*l*__ = ***D***_*f*_*l*__***β***_*f*_*l*__, where ***D***_*f*_*l*__ is a diagonal matrix whose diagonal elements are ***δ***_*f*_*l*__ = (δ_*f*_*l*_1_, δ_*f*_*l*_2_…, δ_*f*_*l*_*t*_), where δ_*f*_*l*_*t*_ is an indicator variable indicating whether the marker effect of locus *f*_*l*_ for trait *t* is zero or not. We use numeric labels “1,” “2,”… , “*z*” to represent all possible combinations for ***δ***_*f*_*l*__, in which case the prior distribution for ***δ***_*f*_*l*__ is: *p*(***δ***_*f*_*l*__ = “i”) = Π_1_*I*(***δ***_*f*_*l*__ = “1”) + Π_2_*I*(***δ***_*f*_*l*__ = “2”) +…+ Π_*l*_*I*(***δ***_*f*_*l*__ = “*z*”) where Π_*i*_ is the prior probability that the vector ***δ***_*f*_*l*__ corresponds to the vector labeled “*i*” and ∑Π_*i*_ = 1. A uniform prior distribution is assigned to Π = (Π_1_, Π_2_, …Π_*l*_) (Cheng et al., 2018b). In multiple-trait BayesB, the prior for ***β***_*f*_*l*__ is a multivariate normal distribution with null mean and locus-specific covariance matrix ***G***_*f*_*l*__, which is assigned an inverse Wishart prior, $W_{t}^{-1}\left(S_{\beta },\nu_{\beta }\right)$. In multiple-trait BayesCΠ, instead of locus-specific covariance matrix ***G***_*f*_*l*__, ***β***_*f*_*l*__ is assumed to follow a multivariate normal prior with null mean and common covariance matrix ***G***, which is assumed to have an inverse Wishart prior distribution, $W^{-1}\left(S_{\beta },\nu_{\beta }\right)$, with degrees of freedom ν_β_ = 4 and scale matrix ***S***_*β*_ such that the prior mean of genetic variance equals half of the phenotypic variance. In single-trait analysis, ***D***_*f*_*l*__, ***G***_*f*_*l*__, and marker effect ***β***_*f*_*l*__ become scalars. The prior of *β*_*f*_*l*__ becomes a univariate normal distribution; the prior of *G*_*f*_*l*__ becomes an inverted chi-square distribution, and *D*_*f*_*l*__ is an indicator variable indicating whether the maker effect is zero or not. In both single-trait and multiple-trait analysis, BayesA and RR-BLUP are just special cases of BayesB and BayesCΠ respectively, where all markers are assumed to have effects on all traits (Fernando and Garrick, 2013). The Bayesian LASSO prior is also included in the multi-class Bayesian Alphabet. In Bayesian LASSO, the multivariate Laplace prior distribution with a null mean is assigned to marker effect vector ***α***_*fl*_ (Gianola and Fernando, 2020) in multiple-trait analysis. In single-trait Bayesian LASSO, the prior for *α*_*f*_*l*__ is a double exponential distribution (Tibshirani, 1996; Gianola, 2013).

### 2.3. Data Analysis

#### 2.3.1. Real Data

Two public datasets are used to evaluate the performance of multi-class Bayesian Alphabet models. The first dataset, which is used to evaluate the single-trait analysis, is composed of genotypic and phenotypic data from Michigan State University Pig Resource Population (MSUPRP) raised at the Michigan State University Swine Teaching and Research Farm, East Lansing,MI (Edwards et al., 2008). After quality control (Duarte et al., 2014), 928 individuals and 42,246 SNPs remain. The trait *13-week tenth rib backfat (mm)* is considered in this analysis. The original data is available at https://msu.edu/~steibelj/JP_files/GBLUP.html. The genome annotation information for the pig dataset used in this paper is obtained from the Ensembl (Rainer et al., 2019) database using the GALLO package (Fonseca et al., 2020). Five annotation regions are identified in the pig dataset, and will be used in our analysis. The number of SNPs in the protein coding, RNA, processed pseudogene, intergenic, and pseudogene regions are 15084, 1840, 107, 24838, and 377, respectively.

The second dataset, which is used to evaluate the multiple-trait analysis, is from the Rice Diversity Panel with 370 *Oryza sativa* individual accessions (Zhao et al., 2011). Three traits *plant height (PH), flowering time in Arkansas (FTA)*, and *panicle number per plant (PN)* are considered. After removing the genotypes missing for these three traits or with minor allele frequency < 0.05, 33,519 SNPs are included in our analysis. The phenotypic and genotypic data are publicly available at http://www.ricediversity.org/. The genome annotation information for the rice dataset is obtained from Ensembl (Rainer et al., 2019) database using the biomart package (Durinck et al., 2009). Four annotation regions are identified in the rice dataset, and will be used in our analysis. The number of SNPs in protein coding, RNA, non-translating CDS, and intergenic regions are 14129, 3, 176, and 19211, respectively.

We identified total 6 genomic annotations: protein coding, processed pseudogene, pseudogene, non-coding RNA, non-translating CDS, and intergenic. According to Howe et al. (2020), the “protein coding” class is comprised of the SNPs within the gene that contains an open reading frame (ORF). In other words, these SNPs may be processed into messenger RNAs (mRNAs) which, after their export to the cytosol, are translated into proteins (Harrow et al., 2009). The “pseudogene” class contains SNPs within the genes that have coding-sequence deficiencies like frameshifts and premature stop codons but resemble protein-coding genes (Howe et al., 2020; Tutar, 2012). The “processed pseudogene” class includes the SNPs in the pseudogene that lack introns and is thought to arise from reverse transcription of messenger RNA followed by reinsertion of DNA into the genome (Howe et al., 2020). The “non-coding RNA” class contains SNPs within RNA that are not translated into a protein (Howe et al., 2020). The “non-translating CDS” class represents SNPs in coding sequence regions that are not translated to a protein (Howe et al., 2020). All other SNPs were allocated to the class “intergenic”.

#### 2.3.2. Simulated Data

To comprehensively compare multi-class Bayesian Alphabet with conventional Bayesian Alphabet for genomic prediction, we conducted simulations based on the real genotypes from Michigan State University Pig Resource Population (MSUPRP) described above (Edwards et al., 2008). The simulation strategies in MacLeod et al. (2016) were applied. 500 QTLs were randomly selected from SNP class “protein coding”, i.e., SNPs with the annotation “protein coding”. In addition, 20 QTLs were randomly selected across the genome. The same QTL positions were used in our simulation. Two correlated traits of heritabilities equal to 0.5 and 0.9 were simulated, where pleiotropic QTL effects were sampled from a multivariate normal distribution with null mean and covariance matrix $G=\left(\begin{array}{cc} 1 & 0.5\\ 0.5 & 1 \end{array}\right)$. The trait of heritability 0.5 was used in our single-trait analysis, and both traits were used in our multiple-trait analysis. There were total 30 different datasets being simulated based on the simulation processes described above.

#### 2.3.3. Cross Validation

The dataset was randomly split into training and validation datasets following an 8:2 ratio for each replicate. 50 replicates and 5 replicates were applied to the real and simulated datasets, respectively. The prediction accuracy was calculated as the mean Pearson correlation between the estimated breeding values and phenotypic records of observations in validation datasets. Conventional and multi-class Bayesian Alphabet methods were compared using RR-BLUP, BayesA, BayesB, BayesCΠ, and Bayesian LASSO priors. In addition to the above five Bayesian methods, an ensemble approach that uses average estimated breeding values across five Bayesian methods, was used to integrate multiple predictions into one summary prediction (Azodi et al., 2019).

The molecular markers were allocated into multiple classes using the genome annotation information. SNP classes were defined using the genome annotation information, i.e., SNPs with the same genome annotation were allocated in one class.

We have implemented these methods in JWAS (Cheng et al., 2018a), an open-source package for single-trait and multiple-trait genome-enabled prediction and analyses. The software tool JWAS offers open-source routines to perform these analyses. The documentation and examples of JWAS can be found at https://github.com/reworkhow/JWAS.jl. MCMC chains of length 100,000 with a burn-in of the first 50,000 iterations were used. The Gelman-Rubin test (Gelman and Rubin, 1992) has been used to verify the convergence of the MCMC chain.

## 3. Result

### 3.1. Simulated Data

**Figure 1:**
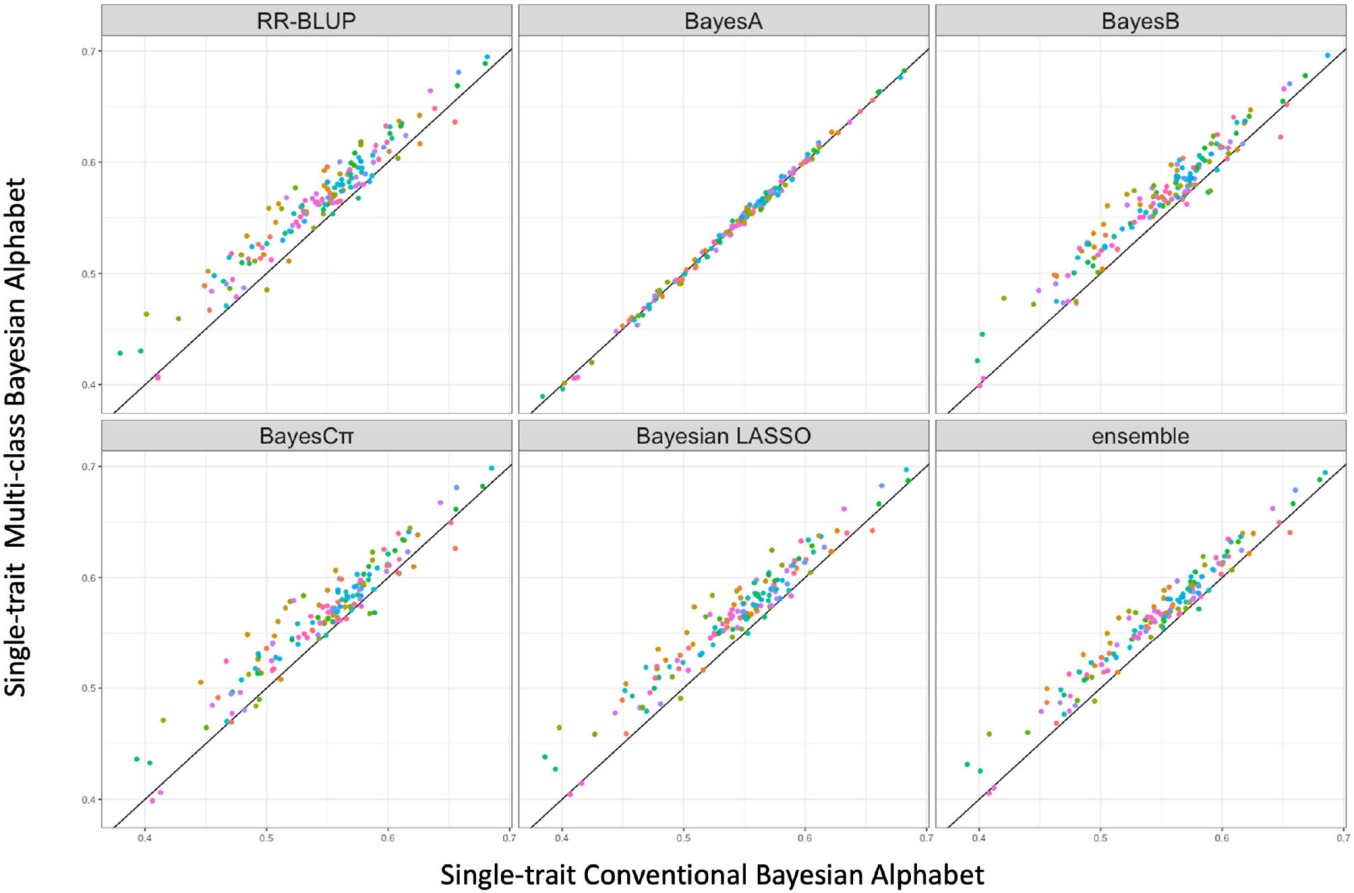
The pairwise predication accuracy comparison between conventional and multi-class Bayesian Alphabet on single-trait simulated data. The 30 simulated datasets were distinguished by color; the x-axis represents the genomic prediction accuracy obtained from conventional Bayesian Alphabet methods; the y-axis represents the genomic prediction accuracy obtained from multi-class Bayesian Alphabet methods; the diagonal line is used for reference such that a dot above the line represents a validation with higher accuracy for multi-class Bayesian Alphabet. Significant differences were found between multi-class Bayesian Alphabet and conventional Bayesian Alphabet with RR-BLUP, BayesB, BayesCπ Bayesian LASSO, and ensemble approach, respectively (*p* < 0.1).

**Figure 2:**
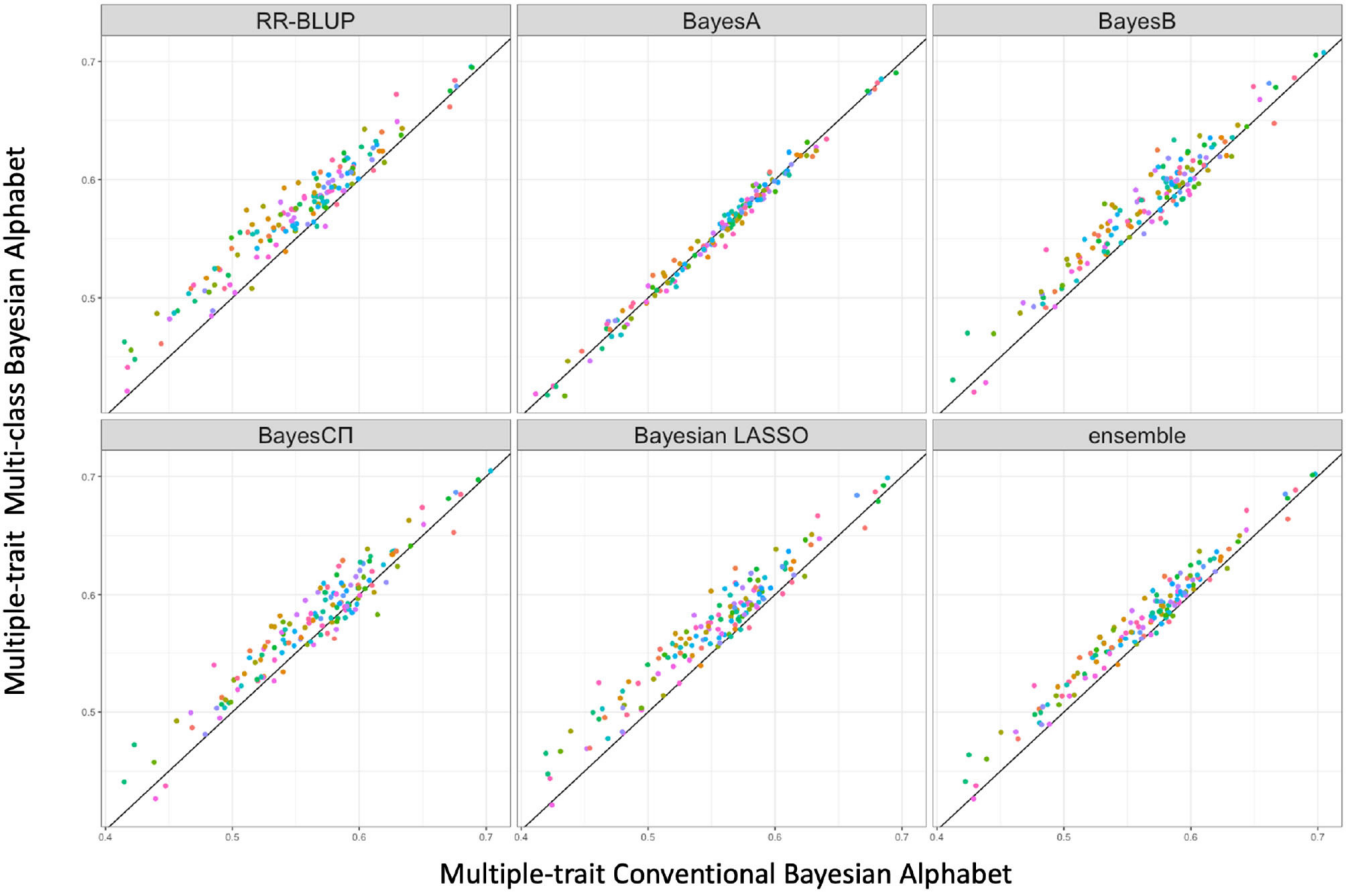
The pairwise predication accuracy comparison between conventional Bayesian Alphabet and multi-class Bayesian Alphabet on multiple-trait simulated data. The 30 simulated datasets were distinguished by color; the x-axis represents the genomic prediction accuracy obtained from conventional Bayesian Alphabet method; the y-axis represents the genomic prediction accuracy obtained from multi-class Bayesian Alphabet method; the diagonal line is used for reference such that a dot above the line represents a validation with higher accuracy for multi-class Bayesian Alphabet. Significant differences were found between multi-class Bayesian Alphabet and conventional Bayesian Alphabet with RR-BLUP, BayesB, BayesCπ Bayesian LASSO, and ensemble approach, respectively (*p* < 0.1).

Multi-class Bayesian Alphabet methods using genome annotation information were performed for both single-trait and multiple-trait prediction on the simulated data. In both single-trait and multiple-trait analysis, 5-fold cross validation was applied on 30 simulated datasets. The comparisons between multi-class and conventional Bayesian Alphabet methods are shown in Table 1 for single-trait analysis and Table 2 for multiple-trait analysis. The pairwise comparisons across all 30 simulated datasets are also shown in Figure 1 for single-trait analysis and Figure 2 for multiple-trait analysis. The 30 simulated datasets are distinguished by color. The paired t-test with a significance level 0.1 is used to declare the significant difference between prediction accuracies from multi-class and conventional Bayesian Alphabet methods.

**Table 1:** Mean prediction accuracy comparison between conventional and multi-class Bayesian Alphabet on single-trait simulated data.

**Method**	**RR-BLUP**	**BayesA**	**BayesB**	**BayesCπ**	**Bayesian LASSO**	**Ensemble**
Conventional	0.542^*^	0.542	0.547^*^	0.547^*^	0.541^*^	0.545^*^
Multi-class	0.563^*^	0.542	0.565^*^	0.565^*^	0.563^*^	0.563^*^

*The comparison of mean prediction accuracy across 150 single-trait validation datasets (30 simulated data × five-fold cross validation) between multi-class Bayesian Alphabet using genome annotation information and conventional Bayesian Alphabet. The paired t-test (p < 0.1) was used to declare the significant difference*.

* *Denotes that significant differences were found between multi-class Bayesian Alphabet and conventional Bayesian Alphabet with RR-BLUP, BayesB, BayesCπ Bayesian LASSO, and ensemble approach, respectively (p < 0.1)*.

**Table 2:** Mean prediction accuracy comparison between conventional and multi-class Bayesian Alphabet on multiple-trait simulated data.

**Method**	**RR-BLUP**	**BayesA**	**BayesB**	**BayesCΠ**	**Bayesian LASSO**	**Ensemble**
Conventional	0.552^*^	0.554	0.565^*^	0.564^*^	0.552^*^	0.561^*^
Multi-class	0.572^*^	0.553	0.578^*^	0.577^*^	0.572^*^	0.575^*^

*The comparison of mean prediction accuracy across 150 multiple-trait validation datasets (30 simulated data × five-fold cross validation) between multi-class Bayesian Alphabet using genome annotation information and conventional Bayesian Alphabet. The paired t-test (p < 0.1) was used to declare the significant difference*.

* *Denotes that significant differences were found between multi-class Baysian Alphabet and conventional Bayesian Alphabet with RR-BLUP, BayesB, BayesCπ, Baysian LASSO, and ensemble approach, respectively (p < 0.1)*.

In the single-trait analysis, significant differences in prediction accuracies were detected between multi-class and conventional Bayesian Alphabet methods with RR-BLUP, BayesB, BayesCπ, Bayesian LASSO priors, and the ensemble approach (*p* < 0.1). In detail, the mean prediction accuracies of multi-class Bayesian Alphabet were higher than conventional Bayesian Alphabet in 30 out of all 30 datasets with RR-BLUP, BayesB, BayesCπ, Bayesian LASSO priors, and ensemble approach. Multi-class Bayesian Alphabet significantly outperforms conventional Bayesian Alphabet in the ensemble approach due to the better performance of multi-class Bayesian Alphabet using these 4 priors.

In the multiple-trait analysis, no significant differences were observed for the higher heritability trait, and results for the lower heritability trait were presented. Overall, higher prediction accuracies were usually observed for the same prior in multiple-trait analysis compared to single-trait analysis. A significant difference in prediction accuracies was detected between multi-class and conventional Bayesian Alphabet methods with RR-BLUP, BayesB, BayesCΠ, Bayesian LASSO prior (*p* < 0.1) as well as the ensemble approach. Similar to single-trait simulation result, the mean prediction accuracies of multi-class Bayesian Alphabet were higher than conventional Bayesian Alphabet in 30 out of all 30 simulated datasets with RR-BLUP, BayesB, BayesCπ Bayesian LASSO priors and the ensemble approach. The simulated data result shows that the multi-class Bayesian Alphabet has the potential to improve the prediction accuracy for both single-trait and multiple-trait analysis.

### 3.2. Real Data

**Figure 3:**
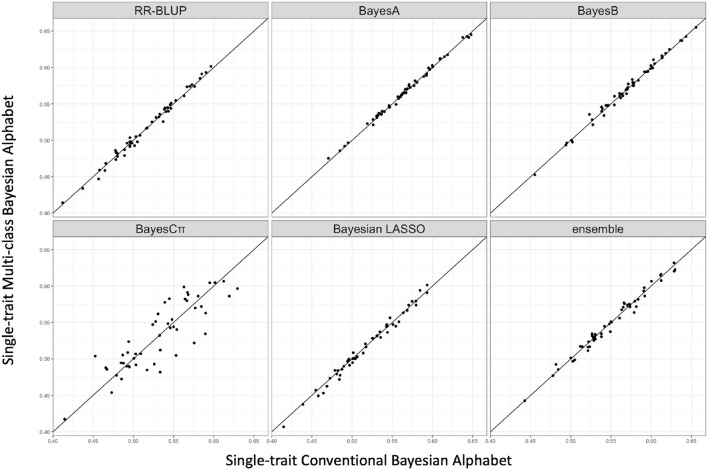
The pairwise prediction accuracy comparisons between conventional Bayesian method and multi-class Bayesian Alphabet using genome annotation classes for the trait *13-week tenth rib backfat (mm)* from pig real data. The x-axis represents the genomic prediction accuracy obtained from conventional Bayesian method; the y-axis represents the genomic prediction accuracy obtained from multi-class Bayesian Alphabet method; the diagonal line is used for reference such that a dot above the line represents a validation with higher accuracy for multi-class Bayesian Alphabet. No significant differences were found (*p* < 0.1).

**Figure 4:**
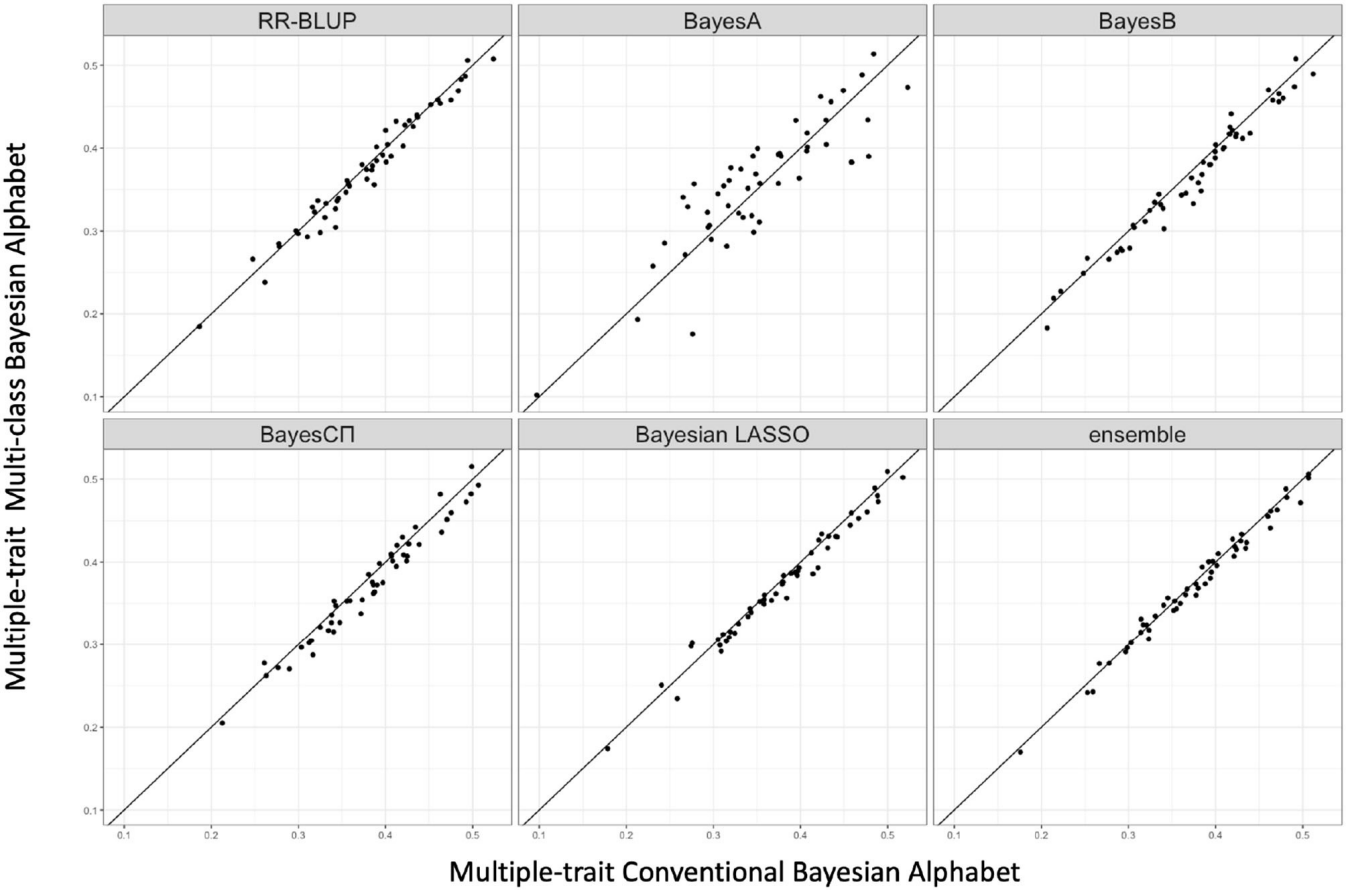
The pairwise accuracy comparisons between conventional Bayesian method and multi-class Bayesian Alphabet using genome annotation classes for the trait *FTA* of rice real data. The x-axis represents the genomic prediction accuracy obtained from conventional Bayesian method; the y-axis represents the genomic prediction accuracy obtained from multi-class Bayesian Alphabet method; the diagonal line is used for reference such that a dot above the line represents a validation with higher accuracy for multi-class Bayesian Alphabet. No significant differences were found (*p* < 0.1).

Multi-class Bayesian Alphabet methods were performed on the pig data (Edwards et al., 2008) for single-trait analysis and the rice data (Zhao et al., 2011) for multiple-trait analysis. In the multiple-trait analysis, three traits *PH, FTA* and *PN* showed similar patterns on the comparison between conventional and multi-class Bayesian Alphabet methods, so only results of trait *FTA* were presented for simplicity. In both single-trait and multiple-trait analysis, 50-fold cross validation was applied. The comparison between multi-class and conventional Bayesian Alphabet methods are shown in Table 3 for single-trait analysis and multiple-trait analysis. The pairwise comparisons across all 50 validation datasets are also shown in Figure 3 for single-trait analysis, and Figure 4 for multiple-trait analysis. The paired t-test with a significance level 0.1 was used to declare the significant difference between prediction accuracies from multi-class and conventional Bayesian Alphabet methods.

**Table 3:** Mean prediction accuracy comparison between conventional and multi-class Bayesian Alphabet for real pig data (single-trait) and real rice data (multiple-trait).

**Data**	**Method**	**RR-BLUP**	**BayesA**	**BayesB**	**BayesCΠ**	**Bayesian LASSO**	**Ensemble**
Pig	Conventional	0.516	0.565	0.568	0.532	0.517	0.550
	Multi-class	0.516	0.565	0.569	0.532	0.516	0.550
Rice	Conventional	0.378	0.353	0.372	0.384	0.378	0.377
	Multi-class	0.374	0.357	0.363	0.375	0.373	0.373

*The comparison of mean prediction accuracy on the trait 13-week tenth rib backfat (mm) from pig data and trait FTA of rice real data across 50 validation datasets between multi-class Bayesian Alphabet using genome annotation information and conventional Bayesian Alphabet. The paired t-test (p < 0.1) was used to declare the significant difference. No significant differences were found between multi-class and conventional Bayesian Alphabet methods for both real pig and rice data (p < 0.1)*.

As shown in Table 3, in both real pig (single-trait) and rice (multiple-trait) data analysis, the prediction accuracies of multi-class Bayesian Alphabet using genome annotation information were not significantly different from conventional Bayesian Alphabet methods for all priors and ensemble approach.

**Figure 5:**
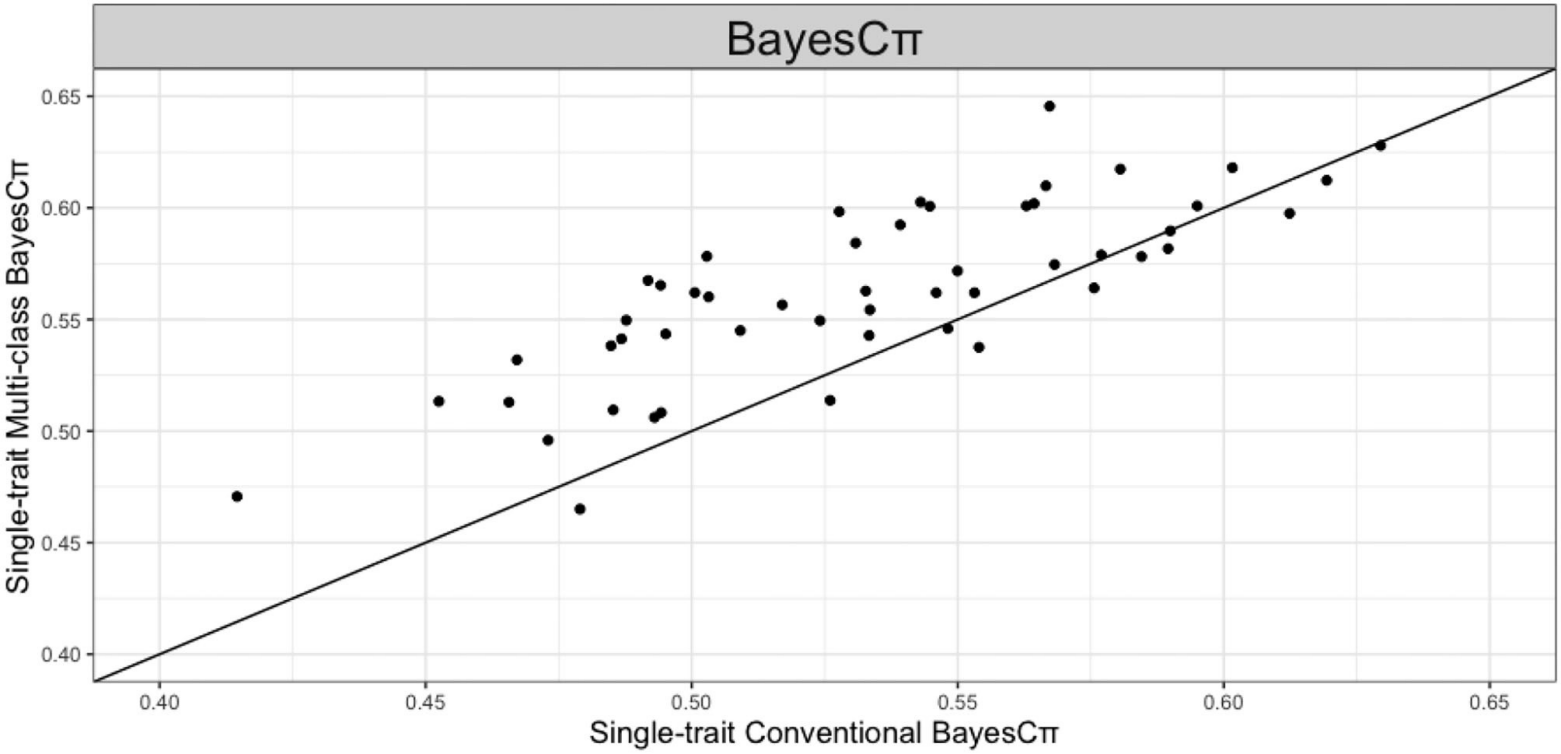
The pairwise prediction accuracy comparisons of conventional BayesCπ and multi-class BayesCπ using chromosome classes for the trait *13-week tenth rib backfat (mm)* of real pig data. The x-axis represents the genomic prediction accuracy obtained from conventional BayesCπ; the y-axis represents the genomic prediction accuracy obtained from multi-class BayesCπ; the diagonal line is used for reference such that a dot above the line represents a validation with higher accuracy for multi-class BayesCπ. Multi-class BayesCπ significantly outperformed the conventional BayesCπ (*p* < 0.1).

We further studied the effect of SNP allocation on prediction accuracy by using other types of known biological information. For example, we allocated SNPs on the same chromosome to the same class such that number of chromosomes classes are fitted in multi-class Bayesian Alphabet methods. As shown in Figure 5, in the real pig (single-trait) data analysis, when BayesCπ prior is used, multi-class Bayesian Alphabet using chromosome classes has significantly higher prediction accuracy than the conventional Bayesian Alphabet (*p* < 0.1). To further understand why higher prediction accuracy is achieved in multi-class BayesCπ using chromosome classes, a genome-wide association study (GWAS) was performed on the same dataset, and one significant signal was detected on chromosome 6 (Chen et al., 2017). Thus, we ran another multi-class Bayesian alphabet analysis by allocating SNPs on chromosome 6 to one class and the remaining to another for a 2-class Bayesian Alphabet analysis. Higher prediction accuracy was observed in this 2-class Bayesian Alphabet analysis. It indicates that assigning SNPs into classes based on GWAS results may be one useful strategy to incorporate biological information.

## 4. Discussion

Most genomic prediction methods usually assume all marker effects share the same prior distribution. This assumption, however, is not biologically meaningful and may potentially reduce the prediction performance when genetic architectures vary across different genomic regions (Speed and Balding, 2014). To address this issue, some methods such as MultiBLUP (Speed and Balding, 2014) and BayesRC (MacLeod et al., 2016) were proposed to allocate markers into different classes, and the superior performances of these methods were observed. Most of these methods, however, focus on single-trait analysis and have limitations in the priors used for marker effects. Thus, in this study, we presented the multi-class Bayesian Alphabet methods, which can perform both single-trait and multiple-trait analysis and provide multiple Bayesian Alphabet priors for markers allocated to different classes.

The effect of allocating markers into different classes on genomic prediction has been studied in some previous studies (Morota et al., 2014; Speed and Balding, 2014; MacLeod et al., 2016; Xu et al., 2020). Different effect-size prior distributions are assigned to molecular markers being split into multiple classes based on genetic architectures. In this paper, we use genome annotation to allocate markers into multiple classes. Note that given the different biological information, the number of classes and markers inside each class might be different. For example, we can use the GWAS results, like Zhang et al. (2014) and Gao et al. (2015), to allocate markers into two classes: one with identified causal variants and another class with the remaining markers.

The comparisons between prediction accuracies from multi-class and conventional Bayesian Alphabet are shown in Tables 1–3. Multi-class Bayesian Alphabet performs consistently equivalent to or better than conventional Bayesian Alphabet in both real and simulated datasets. The different performances of the multi-class Bayesian Alphabet may be caused by the genetic architectures across different genomic regions in the datasets. The methods that allocate markers into different classes outperform the conventional methods because these methods allow different priors on marker effects according to genetic architectures (Speed and Balding, 2014; MacLeod et al., 2016). If genetic architectures are similar across the SNP classes, assigning different priors will not bring significant improvement. For example, in comparisons without much difference between multi-class and conventional methods, e.g., multi-class BayesCπ using genome annotation information in the real pig data analysis, relatively small range (0.0001 to 0.03) for the estimated marker effect variances was observed across SNP classes. However, in comparison with significant differences, e.g., multi-class BayesCπ using chromosome information in the real pig data analysis, relatively large range (0.0001 to 0.15) for the estimated marker effect variances was observed across SNP classes.

Our multi-class Bayesian Alphabet method allows the coexist of the different types of priors in one model. For example, a BayesA prior can be assigned to one SNP class and a BayesCΠ prior to another. In addition, the same marker can be allocated to multiple SNP classes. Compared to other methods that allocate markers into multiple classes, our multi-class Bayesian Alphabet provides more flexibility for model construction given the genetic architectures of the traits of interest and increasing biological knowledge on the genome for both single-trait and multiple-trait analysis. However, a naive comparison among multiple multi-class Bayesian Alphabet methods is computationally intensive. For example, with 6 SNP classes and 5 types of prior, there are 5^6^ possible combinations, and the computational intensity increases dramatically as the number of SNP classes grows. An efficient algorithm to choose biologically meaningful priors for each SNP class, is needed. In addition, biological knowledge generated from other projects may help to narrow down the prior candidates for each SNP class. In our multi-class Bayesian Alphabet methods tested in this paper, where computational intensities are similar to conventional methods, equivalent or better performances are consistently observed. Given that our single-trait and multiple-trait multi-class Bayesian Alphabet methods are biologically meaningful and their implementation is available in an open-source package, we expect it would be widely adopted for genomic prediction.

## Data Availability Statement

The original contributions presented in the study are included in the article/supplementary material, further inquiries can be directed to the corresponding author.

## Author Contributions

HC conceived the study. HC and ZW implemented the method. ZW undertook the analysis and wrote the draft. Both authors contributed to the final version of the manuscript, read, and approved the final manuscript.

## Funding

This work was supported by the United States Department of Agriculture, Agriculture and Food Research Initiative National Institute of Food and Agriculture Competitive Grant Nos. 2018-67015-27957 and 2021-67015-33412.

## Conflict of Interest

The authors declare that the research was conducted in the absence of any commercial or financial relationships that could be construed as a potential conflict of interest.

## Publisher's Note

All claims expressed in this article are solely those of the authors and do not necessarily represent those of their affiliated organizations, or those of the publisher, the editors and the reviewers. Any product that may be evaluated in this article, or claim that may be made by its manufacturer, is not guaranteed or endorsed by the publisher.
